# Supplementary material for: Bacillus thuringiensis Vip1 Functions as a Receptor of Vip2 Toxin for Binary Insecticidal Activity against Holotrichia parallela
Source: Toxins (Basel). 2019 Jul 25;11(8):440. doi: 10.3390/toxins11080440 (PMC6723666; doi:10.3390/toxins11080440)
Supplement: Supplementary file 1 [file toxins-11-00440-s001.pdf]

Supplementary Materials: *Bacillus thuringiensis* Vip1 Functions as a Receptor of Vip2 Toxin for Binary Insecticidal Activity against *Holotrichia parallela*

Jianxun Geng, Jian Jiang, Changlong Shu, Zeyu Wang, Fuping Song, Lili Geng, Jiangyan Duan and Jie Zhang

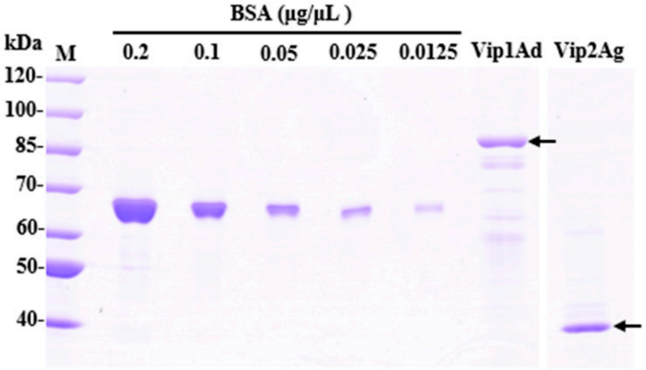

Figure S1. Analysis of Vip1Ad and Vip2Ag expression levels in fermentation broth. The expression of Vip1Ad and Vip2Ag was analyzed by SDS-PAGE. The concentrations of Vip1Ad and Vip2Ag extracts were determined by ImageJ Software using different concentrations of bovine serum albumin (BSA) as a standard. Arrowheads indicate the expressed Vip1Ad and Vip2Ag.

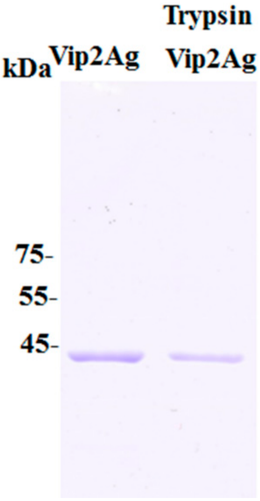

Figure S2. SDS-PAGE analysis of Vip2Ag was digested by trypsin.

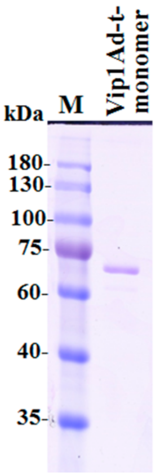

Figure S3. SDS-PAGE analysis of Vip1Ad-t-monomer was purified by Superdex-75 gel filtration chromatography.

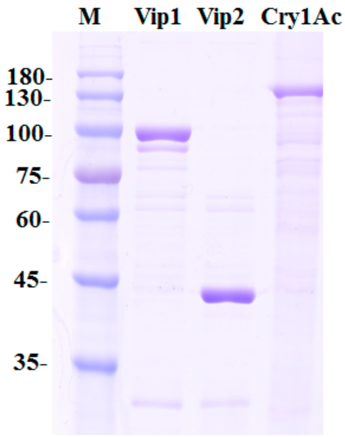

Figure S4. Comparison of Vip1Ad, Vip2Ag and Cry1Ac protein expression level.
